# Substrate-Induced Structural Dynamics and Evolutionary Linkage of Siderophore-Iron ABC Transporters of *Mycobacterium tuberculosis*

**DOI:** 10.3390/medicina60111891

**Published:** 2024-11-18

**Authors:** Aisha Farhana, Abdullah Alsrhani, Hasan Ejaz, Muharib Alruwaili, Ayman A. M. Alameen, Emad Manni, Zafar Rasheed, Yusuf Saleem Khan

**Affiliations:** 1 Department of Clinical Laboratory Sciences, College of Applied Medical Sciences, Jouf University, Sakaka 72388, Aljouf, Saudi Arabia; afalserhani@ju.edu.sa (A.A.); hetariq@ju.edu.sa (H.E.); mfalrwaili@ju.edu.sa (M.A.); aaalameen@ju.edu.sa (A.A.M.A.); emena@ju.edu.sa (E.M.); 2 Department of Pathology, College of Medicine, Qassim University, Buraidah 51452, Qassim, Saudi Arabia; zafarrasheed@qu.edu.sa; 3 Department of Anatomy, College of Medicine, University of Hail, Hail 55476, Hail, Saudi Arabia

**Keywords:** *Mycobacterium tuberculosis*, siderophore-iron uptake, ABC transporters, Rv1348 (IrtA) and Rv2895c (IrtB), evolutionary linkage of transporters, drug targeting

## Abstract

*Background and Objective*: ATP-binding cassette (ABC) transporters are prominent drug targets due to their highly efficient trafficking capabilities and their significant physiological and clinical roles. Gaining insight into their biophysical and biomechanistic properties is crucial to maximize their pharmacological potential. *Materials and Methods*: In this study, we present the biochemical and biophysical characterization, and phylogenetic analysis of the domains of *Mycobacterium tuberculosis* (*M. tuberculosis*) ABC transporters: the exporter Rv1348 (IrtA) and the importer system Rv1349-Rv2895c (IrtB-Rv2895c), both involved in siderophore-mediated iron uptake. *Results*: Our findings reveal that the substrate-binding domain (SBD) of IrtA functions as an active monomer, while Rv2895c, which facilitates the uptake of siderophore-bound iron, exists in a dynamic equilibrium between dimeric and monomeric forms. Furthermore, ATP binding induces the dimerization of the ATPase domains in both IrtA (ATPase I) and IrtB (ATPaseII), but only the ATPase domain of IrtA (ATPase I) is active independently. We also analyzed the stability of substrate binding to the domains of the two transporters across varying temperature and pH ranges, revealing significant shifts in their activity under different conditions. Our study highlights the conformational changes that accompany substrate interaction with the transporter domains, providing insights into the fundamental mechanism required for the translocation of siderophore to the extracytoplasmic milieu by IrtB and, subsequently, import of their ferrated forms by the IrtB-Rv2895c complex. Phylogenetic analyses based on ATPase domains reveal that IrtA shares features with both archaeal and eukaryotic transporters, while IrtB is unique to mycobacterial species. *Conclusions*: Together, these findings provide valuable insights, which could accelerate the development of intervention strategies for this critical pathway pivotal in the progression of *M. tuberculosis* infection.

## 1. Introduction

*Mycobacterium tuberculosis* (*Mtb*) is among the pathogens that cause chronic and fatal illness. Approximately 1.3 million people die from tuberculosis each year despite a great deal of research and therapeutic efforts [1]. Current therapeutic interventions are impeded by long treatment regimens, which frequently result in non-compliance and the establishment of multidrug-resistant strains [2]. Bacillus Calmette-Guérin (BCG), the only vaccine currently available, provides protection that varies from very effective to almost ineffective across different populations [3].

Acquisition of essential micronutrients and ligands is a critical strategy for the survival and persistence of intracellular pathogens like mycobacteria and several other pathogenic bacteria [4,5,6]. Detailed analyses of molecular systems involved in nutrient trafficking, including ATP-binding cassette (ABC) transporters, are crucial in advancing the development of novel anti-mycobacterial therapies [7,8]. ABC transporters, specifically those involved in siderophore-based ATP-dependent iron transport, play a vital role in replenishing iron during active *M. tuberculosis* infection, underscoring their significance within the iron-limited environment of the host [9,10,11,12,13]. Among these, the synergistic siderophore export and iron-laden import system mediated by the ABC transporters IrtA and IrtB-Rv2895c is essential for pathogen survival, as demonstrated in mouse infection models. This makes these iron-siderophore transporters appealing targets for drug development [11,12,14].

ABC transporters are characterized by unique signature motifs, such as the LSGGQMRRVVLAGLL and LSGGELQRLALAAAL sequences and ATP-binding motifs, i.e., GXXXXGKT and GXXXXGKS within their protein sequences, enabling the identification of ABC transporters. The importance of the several ABC transporters as drug targets has been extensively evaluated, and their repertoire has been successfully validated in in vitro and in vivo studies [15,16,17,18,19,20]. ABC transporters constitute one of the largest and most diverse protein super-families, mediating ATP-dependent solute transport and maintaining their intracellular concentrations [21]. They are therefore central to cellular functions in both eukaryotes and prokaryotes [22,23]. A key distinction between eukaryotic (EK-type) and prokaryotic (PK-type) transporters lies in their gene and domain organization [24]. EK-type transporters are frequently found as domains inside a single polypeptide, while PK-type transporters are usually composed of separate components [22]. While PK-type transporters predominantly act as importers, obtaining necessary components vital to cell viability, EK-type transporters largely facilitate the efflux of different compounds and drugs, qualifying them as exporters [25]. ABC transporters are crucial targets for therapeutic treatments because of their clinical significance as multidrug efflux pumps and their function in the inflow of vital nutrients [26,27].

A coordinated interaction between substrate recognition, binding to the substrate-interacting domain, and the subsequent translocation through the transmembrane permease is required for ABC transporters to transport substrates. This unidirectional transport process is driven by the ATPase activity of the nucleotide-binding domain (NBD) present on the cytoplasmic side [21,25]. These systems are precisely calibrated to serve as composite transport channels appropriate for certain molecules and, occasionally, several different kinds of molecules [28]. Considered a hallmark of ABC transporters, NBD is conserved across archaeal, bacterial, and eukaryotic species [29,30,31]. However, there is less sequence similarity and structural homology among the transmembrane permeases and substrate-binding domains, which dictate subfamily-specific functions. Due to the structural diversity of substrates across species and the varied mechanical and functional characteristics of these domains, transmembrane (TM) and substrate-binding domains undergo faster, substrate-dependent evolution and divergence compared to the ABC hydrolyzing subunits [32]. The transporters of the efflux system branch away from that of the influx transporters [33]. Given that efflux systems are more highly developed and widespread in prokaryotes compared to influx systems, it has been hypothesized that influx systems may have evolved from efflux mechanisms.

Mechanistic investigations of transporter proteins reveal that during transport, substrate-bound and unbound forms transition between conformations with high and low affinity. These conformational changes, which are triggered by substrate binding and/or ATP hydrolysis, are essential for controlling solute transport across membranes [34]. The intricate network and signal relay among the substrate-binding, transmembrane, and ATPase domains are essential for the functioning of these transporters. About 37 complete (including at least one TM and one ABC domain) and incomplete ABC transporters have been found based on structural and sequence similarities in *M. tuberculosis*, where genes encoding ABC transporters make up almost 2.5% of the genome [35]. Despite their significance, the study of membrane transporters is still challenging, mostly because of the difficulties in extracting recombinant integral membrane proteins while preserving their stability and activity.

In this study, we analyze the substrate-induced biochemical and biophysical alterations of the subunit domains in the IrtA and IrtB-Rv2895c systems, which are intrinsic to the function of these transporter proteins. Additionally, a phylogenetic analysis based on the conserved NBD domain was conducted to explore the evolutionary relationships and relatedness of these two transporters.

## 2. Materials and Methods

**Figure 1:**
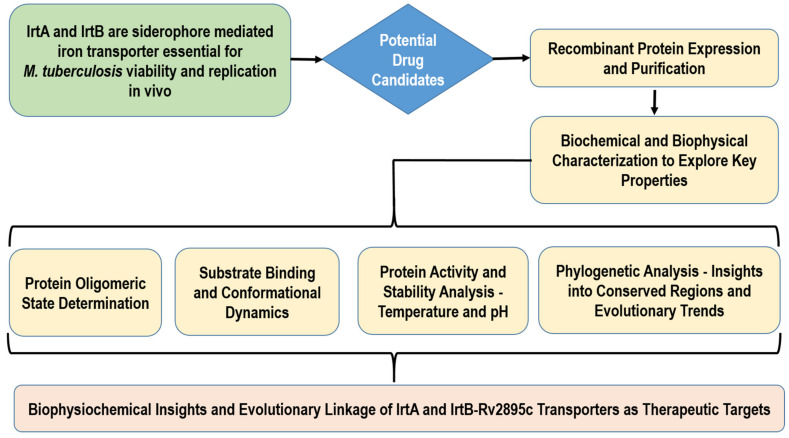
The above flowchart outlines the research methodology for comprehensive characterization of IrtA and IrtB-Rv2895c transporter proteins aiming to elucidate their biophysicochemical properties and explore their evolutionary relationships.

The following flowchart shows the research method for carrying out biophysical and biochemical characterization as well as the evaluation of evolutionary linkage for IrtA and IrtB-Rv2895c (Figure 1). In addition to offering important insights into drug development, the procedure seeks to further our understanding of the therapeutic potential of transporter proteins.

### 2.1. Protein Expression and Purification

*IrtA* was cloned into the pET23a expression vector and *IrtB*, *SBD*, and *Rv2895c* were cloned into pET28a, as described earlier [12]. The ATPase domains of *IrtA* (ATPase I) and *IrtB* (ATPase II) were also cloned in pET23a at *Nde*I/*Hin*dIII sites using primer pairs:

5′-CCCATATGCGGCACCTGCAAGTCACACTCGAC-3′ and

5′-CGAAGCTTTCGGGTGCCGTCCTGCGCTGCG-3′ for ATPase I, and

5′-CCCATATGGCACCGGTCATGGTGGCCGGGTC-3′ and

5′-CGAAGCTTCTCGGCGAGGATCTGCCACTCG-3′ for ATPase II.

**Figure 2:**
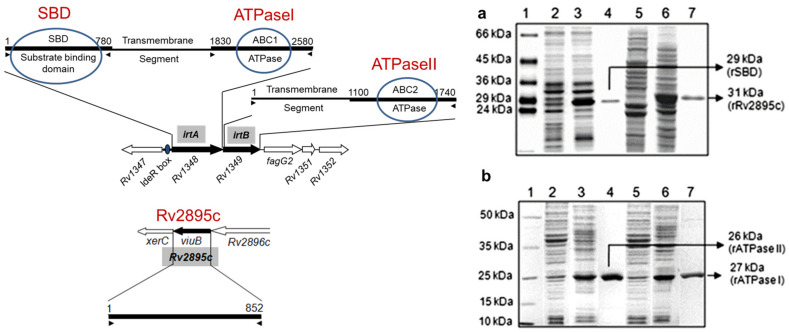
Protein expression and purification. SDS PAGE analyses were carried out for the total cell lystes of *E. coli* BL-21 DE3 cod+ harboring (**a**) rSBD and rRv2895c and (**b**) is rATPase I, and rATPase II plasmid constructs. In both a and b, lane 1 is the protein marker. Lanes 2 and 5 are uninduced samples, lanes 3 and 6 are samples induced for 3 h, and lanes 4 and 7 are purified recombinant proteins. In (**a**), lanes 2–4 correspond to SBD and lanes 5–7 correspond to rRv2895c. In (**b**), lanes 2–4 correspond to rATPase I, and lanes 5–7 for rATPase I. The molecular size of each protein is indicated with an arrow. (Left) The panel on the left shows a schematic representation of the genes and domains on the *M. tuberculosis* genome (adapted from Farhana et al., 2008, ref. [12]).

The rATPase I and rATPase II domains and SBD and Rv2895c proteins were expressed and purified, as shown in Figure 2a,b. The expression and purification procedures of IrtA and IrtB integral membrane proteins are provided elsewhere [12]. Briefly, for the expression and purification of rATPase I, rATPase II domains, rSBD and rRv2895c, *E. coli* BL21 cod+ cells were transformed with the recombinant constructs, and the proteins were purified as described previously [12]. To check the protein expression and subsequently confirm the purity of each sample, western blotting of the induced samples and eluates was done using anti-His and protein-specific antibodies. The protein concentrations were measured using a BCA estimation kit. The proteins were finally concentrated using Amicon concentrators to a final concentration of 1 mg/mL.

### 2.2. Analytical Size Exclusion Chromatography

Analytical gel filtration was performed to monitor the oligomeric state of the recombinant proteins, namely, independently expressed SBD, ATPase domains (ATPase I and ATPase II), and Rv2895c protein. Gel chromatography was carried out for the unbound form and after incubation with equimolar concentration of substrate carboxymycobactin (cMyco) for rSBD; ferricarboxymycobactin (Fe-cMyco) for rRv2895c; and 1 mM ATP for rATPase I and rATPase II domains) for 30 min at room temperature (RT). Gel filtration was carried out on BioRad FPLC using Suparose-200 column for rSBD and rRv2895c and Supadex-75 column (BioRad, Hercules, California, USA) for rATPase I and rATPase II with 30 mM Tris (pH 8.0), and 150 mM NaCl as running buffer. The recombinant proteins were loaded at a concentration of 1 mg/mL in the presence of 1 mM DTT for SBD and the two ATPase domains (rATPase I and rATPase II) and without DTT for Rv2895c. The elution volume of the proteins was recorded from the start of the sample application to the apex of each elution peak. The molecular size of each protein was calculated in terms of the log elution volumes of standard protein molecular size markers loaded for each run. Blue Dextran was used to determine void volume.

### 2.3. Circular Dichroism

Far-UV CD spectroscopy of the proteins was carried out in unbound and substrate-bound forms to assess substrate-induced secondary structure changes for all four domains. The binding of cMyco to rSBD, Fe-cMyco to rRv2895c, and ATP to the two ATPase domains, rATPase I and rATPase II, and the consequent change in the structure was monitored on JASCO J-810 CD spectropolarimeter equipped with thermocontroller, with a cuvette path length of 1 mm at 25 °C. The CD spectra were monitored at a protein concentration of 1 mg/mL in 30 mM Tris-HCl, pH 7.5 for each analysis. The protein-to-substrate concentration was taken at a molar ratio of 1:1. Four scans were averaged to get smooth spectra and corrected for the buffer baseline. Analysis of the results was carried out using the K2D2 software (version 2) in all experiments.

### 2.4. Effect of pH and Temperature on Protein Activity

The effect of pH and temperature on substrate binding and activity of the rSBD, rRv2895c, and the two rATPase domains was determined at pH ranging from 4.0 to 9.0 and temperature from 20 °C to 90 °C. pH dependence was analyzed by carrying out substrate binding and activity at 25 °C for 30 min in assay buffer (30 mM Tris, 100 mM NaCl and 1% glycerol for rSBD and rRv2895c; 50 mM HEPES/KOH, 10 mM MgCl_2_, 1 mM EGTA, 2.8 mM β–ME and 30µCi^γ^P-32 ATP equivalent to 1 mM ATP, for rATPase I and rATPase II). The pH values of the buffer were adjusted to 4.0, 5.0, 6.0, 7.0, 8.0, and 9.0 using Tris-HCl or HEPES/KOH. Similarly, temperature stability was assessed by pre-incubating the proteins for 30 min at different temperatures in the above-mentioned buffers for each protein, followed by the addition of substrates and subsequent incubation for 30 min. The optimum temperature of the reaction was determined by varying the assay temperatures from 20 °C to 90 °C with 5 °C or 10 °C intervals. Fluorimetry was carried out for each reaction to ascertain the effects of pH and temperature on substrate binding to rSBD and Rv2895c, whereas substrate binding and activity of rATPase I and rATPase II were assessed by methods described below.

### 2.5. ATP Binding/ATPase Assay

The ATP binding to the recombinant ATPase domain proteins, rATPase I and rATPase II was carried out as mentioned above for pH and temperature optimization. The bound ATP was UV crosslinked to the rATPase protein as previously described [36]. The reactions were stopped by adding 1X SDS loading buffer and fractionated on 10% SDS-PAGE. The binding was assessed with radioactive ATP (α-P32ATP), which was visualized by autoradiography. On the other hand, ATPase activity of the two domains was assessed by monitoring the inorganic phosphate release as mentioned earlier [12]. The ratio of inorganic phosphate released in the presence of ATPase domain proteins to the basal ATP hydrolysis in the absence of protein was plotted as the percentage activity of the proteins. SD ± mean was calculated from three independent experiments.

### 2.6. Fluorimetry

The pH and temperature stability of cMyco binding to rSBD and Fe-cMyco to rRv2895c was determined by monitoring the intrinsic tryptophan fluorescence of the protein-bound forms at different temperatures and pH. Fluorescence spectra of individual samples were recorded, in the presence and absence of substrate using Perkin Elmer LS50B luminescence spectrometer. The change in the maximum fluorescence intensity was determined by excitation at 295 nm and emission recorded from 305–440 nm. It requires mentioning that the intrinsic fluorescence of either cMyco or Fe-cMyco does not overlap with that of tryptophan fluorescence of the two proteins, namely rSBD and rRv2895c. Each experiment was done in triplicate. The change in the fluorescence intensity of the substrate-bound forms compared to the unbound forms (δF/F) at a particular pH or temperature was evaluated to deduce the binding efficiency.

### 2.7. Phylogenetic Analysis

The conserved domains NBDs from IrtA and IrtB served as the query sequence for BALLAST to look for similar sequences within the *Mtb* genome and other organisms [37]. BALLAST takes a reference sequence and searches the protein sequence database (SwissProt + SpTrEMBL + PDB) for homologs using BlastP (E < 0.1) and uses the output to delineate the segments more conserved than their flanking regions [38]. A conservation profile, thus, generated from these local maximum conserved segments (LMSs) is used to rescore blast results. The conserved segments were subsequently used as soft anchors to guide a high-quality multiple alignment of potential homologs using the Dbclustal [39]. The multiple alignment constructed was refined by RASCAL [40] and then validated by LEON [41] to remove any false homologs or unrelated sequences from the alignment. This high-quality validated alignment was clustered using the Secator [42] algorithm that automatically clusters the nodes of a phylogenetic tree to define probable functional subfamilies.

## 3. Results

### 3.1. Recombinant Proteins for Functional Characterization

Recombinant proteins, IrtA, IrtB, ATPaseI, ATPase II, SBD, and Rv2895c, were procured through cloning, followed by an expression in the *E. coli* BL-21 codon+ cells and subsequent purification, as described in Material and Methods Section 2.1. The results of the expression and purification are provided in Figure 2a,b. In Figure 2a, lane 3 shows induced rSBD, while lane 4 is the purified recombinant SBD protein, lane 6 is induced rRv2895c, and lane 7 is the purified recombinant Rv2895c protein. In Figure 2b, lane 3 shows induced ATPase II, while lane 4 is the purified recombinant ATPase II protein, lane 6 is induced recombinant ATPase I, and lane 7 is the purified recombinant ATPase II protein. The left panel in Figure 2 illustrates the representation of the cloned genes and domains on the *M. tuberculosis* genome.

### 3.2. Dynamic Equilibrium and Substrate Binding of Transporter Domains

**Figure 3:**
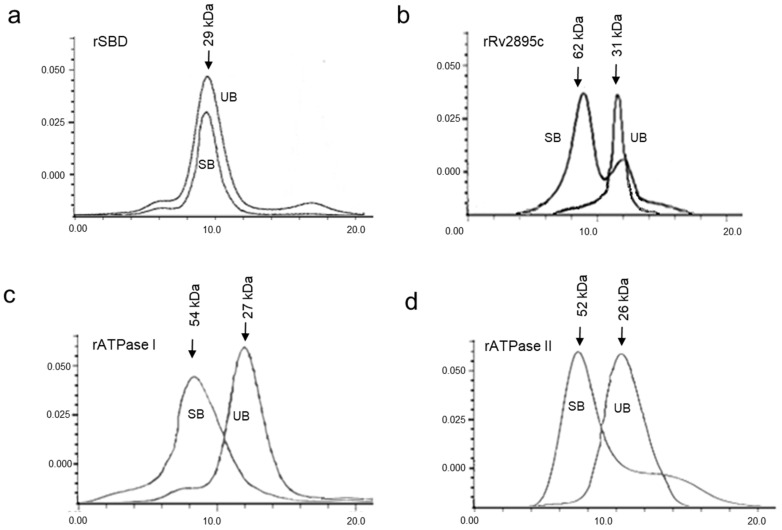
Gel filtration chromatography of native and substrate-bound domains. Elution profiles of 1 mg/mL solutions of unbound and substrate-bound (**a**) rSBD domain, (**b**) rRv2895c proteins fractionated over Superdex-200 column, and (**c**) rATPase I and (**d**) rATPase II fractionated over Sephadex-75. The elution profile generated by the unbound and the substrate-bound (cMyco for rSBD and Fe-cMyco for rRv2895c) protein is labeled as UB (unbound) and SB (substrate-bound), respectively. The molecular size of all the proteins was calculated by comparing them with protein molecular weight standards fractionated on the same column before the samples were loaded.

The functional oligomeric state of substrate-bound siderophore-interacting domains of the two transporters, IrtA and Rv2895c-IrtB, was assessed by gel filtration chromatography. We observed that SBD remains active as a monomer (Figure 3a), whereas the siderophore-bound rRv2895c exists in a dynamic equilibrium between monomeric and dimeric states (Figure 3b). The analysis of the purified domains, both in their unbound and substrate-bound forms, revealed that cMyco binding to the SBD does not induce oligomerization. The overlapping gel elution patterns of the bound and unbound forms show that they are predominantly monomers (Figure 3a). In contrast, Fe-cMyco bound to Rv2895c shows a significant presence of both monomeric and dimeric forms (Figure 3b). This implies that a temporary substrate binding generated a dynamic balance between the two forms. However, the protein is predominantly a dimer when bound to Fe-cMyco. The elution volume of the substrate-bound or unbound proteins was calibrated on a Superose 12 column using molecular size markers to ascertain the molecular weight of the oligomeric forms.

### 3.3. Dimerization of the ATPase Domains of IrtA (ATPase I) and IrtB (ATPase II) Stimulated by the ATP

The active form of ATPase domains, which facilitates the catalytic cycle of ABC transporters, is typically dimeric across almost all ABC transporters. To ascertain the oligomeric state of the ATPase domains of IrtA and IrtB, namely ATPase I and ATPase II, respectively, we performed analytical gel filtration in the presence and absence of ATP (Figure 3c,d). It is interesting to note that the addition of Na-orthovanadate, which prevents ATP hydrolysis, was the only way to capture the dimeric form of ATPase I (the ATPase domain from IrtA). This suggests that dimerization is the mechanism through which the hydrolytic activity of ATPase I is activated. Compared to the monomeric form, the peak arising from ATP-dependent dimerization is somewhat less intense (Figure 3c), suggesting minimal hydrolytic activity that leads to subsequent monomerization of ATPase I. However, the concentration of the monomeric form generated after ATP hydrolysis is likely below the threshold detection limit and, therefore, does not appear as a second peak (Figure 3c). Conversely, ATPase II (the ATPase belonging to IrtB) dimers were captured as stable dimers (Figure 3d) even in the absence of Na-orthovanadate, suggesting that the protein, although dimerizing in the presence of ATP, either remains inactive and unable to hydrolyze its substrate or is locked in the dimeric conformation after hydrolysis, a hypothesis that requires further investigation (Figure 3d). It is therefore inferred that rATPase II is activated upon ATP binding and is likely responsible for initiating the activation of the IrtB exporter protein. On the other hand, ATPase I of IrtA may require an additional signal, besides ATP binding, for its activation.

### 3.4. Conformational Changes Accompanying the Complexation Between the Substrate and the Transporter Domains

**Figure 4:**
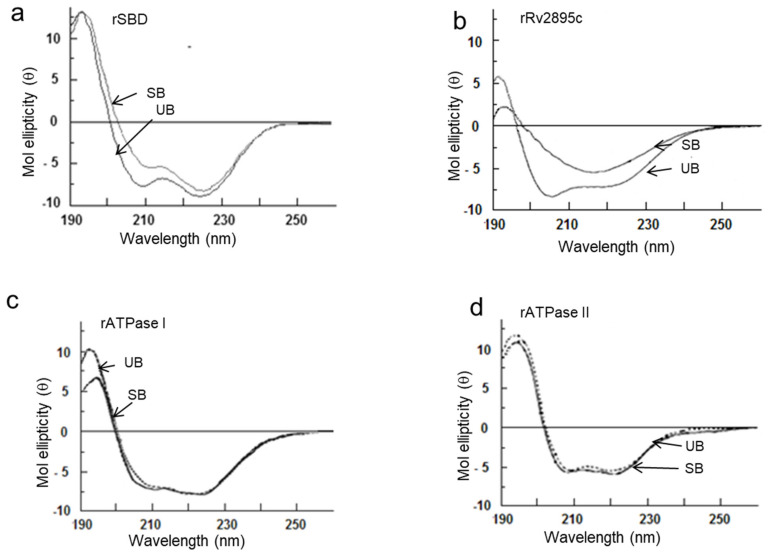
Only rSBD and rRv2895c show substrate-induced secondary structure in far-UV CD spectra, indicating substrate-induced secondary structural changes. CD analyses of (**a**) rSBD, (**b**) rRv2895c, (**c**) rATPase I, and (**d**) rATPAse II were carried out in unbound (UB) and substrate-bound (SB) forms. The spectra shown here are resultant after normalizing with buffer and ligand spectra. Five readings were taken in each case.

Transporter proteins undergo concerted structural changes that modulate the movement of substrates across the membrane. To understand the mechanisms involved in the transport process of the two transporter systems, we conducted circular dichroism (CD) analysis of the independently expressed domains in both substrate-bound and unbound forms. The intense ellipticity at 208 nm and 222 nm in the spectra of the apo forms of rSBD andrRv2895c indicates a significant α-helical character (Figure 4a,b). The binding of cMyco to SBD reduces the percentage of helicity at 208 nm, with a positive amplitude at 196 nm, indicating the presence of unstructured or highly flexible regions, which remains unchanged (Figure 4a). In contrast, the conformation of Rv2895c undergoes significant alteration, shifting from a 90% α-helical structure in its apo form to mostly β-sheet in its Fe-cMyco bound form, along with a decrease in the intensity of flexible coils (Figure 4b). The observed minima at 218 nm and reduced ellipticity at 196 nm suggest a more ordered β-sheet conformation of the protein upon Fe-cMyco binding (Figure 4b). Compared to the siderophore interaction domains, the binding of ATP to either rATPase I or rATPase II does not induce major structural perturbations, as shown in Figure 4c,d. In both substrate-bound and unbound forms, both proteins exhibit strong negative ellipticity around 208 and 222 nm, which is suggestive of a primarily α-helical structure. However, as shown by the 11.7% increase in ellipticity at 208 nm and the small decrease at 196 nm, ATP binding to ATPase I results in a conformational shift that increases the amount of stable α-helix and decreases the amount of unstructured or flexible protein (Figure 4c). Additionally, ATP interaction does not substantially change the confirmation of the ATPase II domain, as seen in Figure 4d, which shows a modest decrease in unstructured areas at 196 nm and a 2% rise in negativity at 222 nm. (Figure 4d). Overall, in comparison to rSBD, rATPase I, or rATPase II domains, rRv2895c exhibits notable structural alterations upon substrate interaction.

### 3.5. pH and Temperature Variation and Its Impact on Transporter Activity

**Figure 5:**
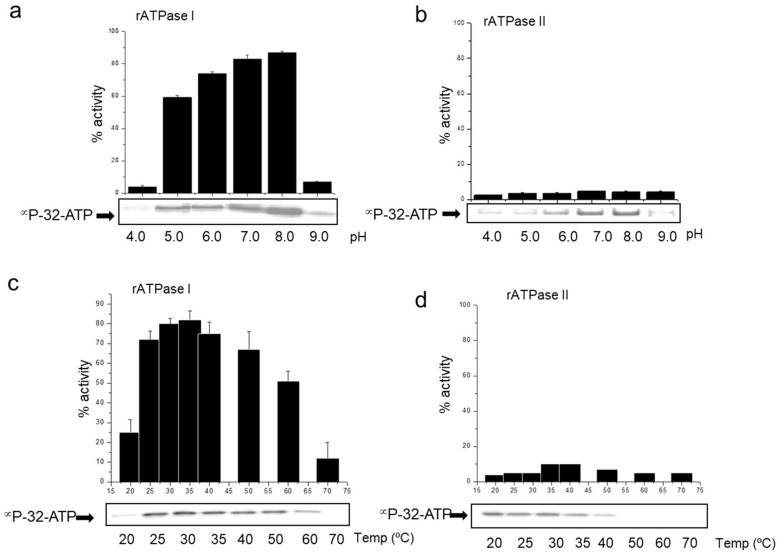
ATPase I but not ATPase II activity is dependent on pH and temperature. The (**a**,**b**) pH and (**c,d**) temperature stability of ATP binding and hydrolysis of (**a**,**c**) ATPaseI and (**b**,**d**) ATPaseII is shown. ATP hydrolyzing activity of the two proteins is represented as a histogram indicating % activity as a function of pH or temperature whereas ATP binding is shown by αP^32^-ATP autoradiogram as panel below the activity graph for each set. The temperature dependence was monitored using buffer at pH 8.0 and pH dependence was monitored keeping the temperature constant at 30 °C.

Variations in the temperature and pH can have a substantial impact on the transporter activities, leading to shifts in substrate binding and translocation. We analyzed the pH and temperature stability of the transporter domain-substrate complexes of the Rv2895c-IrtB and IrtA systems in order to investigate the pH and temperature dependency. Substrate binding and transporter activity were assessed across temperatures ranging from 20 °C to 70 °C (in 10 °C increments) and pH levels from 4.0 to 9.0 (in 1.0 pH unit increments). ATP binding to the ATPase I domain and its hydrolysis demonstrated significant resistance to temperature (Figure 5a) and pH (Figure 5c) changes. This resistance is evident from the substantial ATP binding (as shown in the autoradiogram panel) and its subsequent hydrolysis (as shown in the bar graphs) observed at temperatures up to 60 °C and pH between 5.0 and 8.0. ATPase I has the maximum amounts of ATP binding and hydrolysis at 30 °C and pH 8.0, indicating a temperature and pH-dependent action. On the other hand, ATP binding to ATPase II is essentially insignificant as a function of pH (Figure 5b) and temperature (Figure 5d), suggesting that these variables have no impact on ATPase II activity. This result is consistent with gel filtration, which showed that dimeric forms produced with ATP were stable but hydrolytically inactive (Figure 3d).

**Figure 6:**
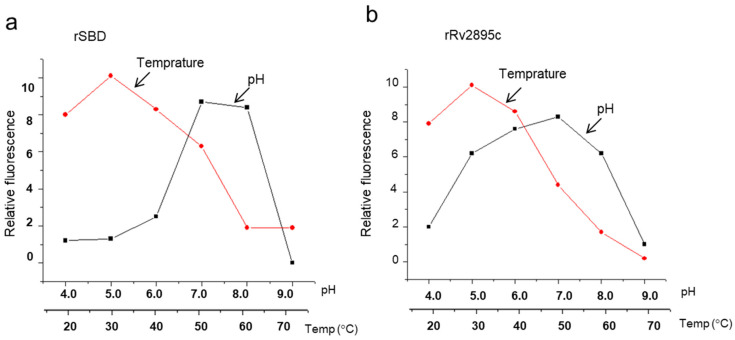
pH and temperature profiling of substrate binding to SBD and Rv2895c. Fluorescence quenching, indicative of substrate binding, was evaluated across varying pH levels and temperatures for (**a**) SBD and (**b**) Rv2895c. The curves with circular data points represent the temperature dependence of substrate binding, while those with square data points illustrate pH dependence. cMyco was used as the substrate for SBD, and Fe-cMyco for Rv2895c. The graph presented is representative of three independent experiments, showing relative fluorescence (δF/F) as a function of pH or temperature.

Fluorescence quenching of the cMyco-bound form in comparison to the unbound form demonstrated the rSBD’s limited pH stability range of 7.0 to 8.0, indicating its substrate-binding capacity. The compound remained stable up to 50 °C, after which there was a noticeable drop in fluorescence (Figure 6a). The ideal temperature for stability was determined to be 30 °C (Figure 6a). Contrarily, rRv2895c showed a bell-shaped curve with an apparent plateau between pH 5.0 and 8.0, suggesting a wider pH stability range for substrate-bound forms (Figure 6b). Nevertheless, the stability of the rRv2895c-bound complex was more sensitive to temperature alterations, peaking at 30 °C followed by a decline as the temperature increased. Further increase in the hampered complex formation, as indicated by a drop in relative fluorescence quenching of the Fe-cMyco-bound forms (Figure 6b). These findings demonstrate that the siderophore-binding and ATPase domains require optimal pH and temperature conditions in order to regulate the transport process.

### 3.6. Evolutionary Origins of Mtb IrtA and IrtB

**Figure 7:**
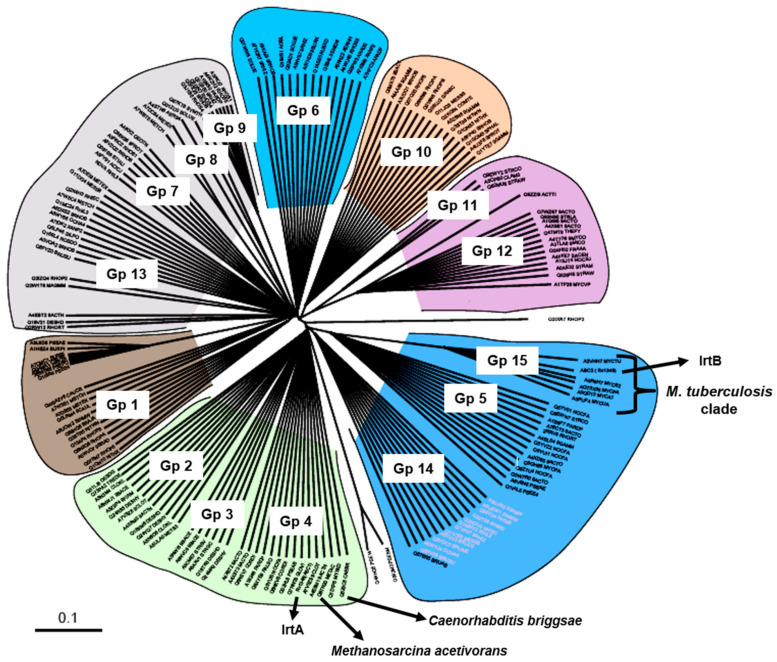
Phylogenetic tree indicating protein function based evolutionary relationship of IrtA and IrtB. A phylogenetic tree was constructed to illustrate the evolutionary relationships of IrtA and IrtB based on their protein functions. The tree comprises 15 groups, labeled Group 1 (Gp1) through Group 15 (Gp15). Group 4 (Gp4) includes *M. tuberculosis* IrtA along with functionally related proteins from archaea and eukaryotes. Group 15 (Gp15) contains IrtB and transporters that are exclusive to the mycobacterial clade.

We previously demonstrated the function and mechanisms of IrtA and IrtB in the siderophore-mediated iron transport process of *M. tuberculosis* [12]. For a better understanding of the roles of these transporters and their evolutionary linkage, we conducted a phylogenetic analysis using the nucleotide-binding domain (NBD) of IrtB as the seeding input. The analysis identified that proteins related to the IrtB NBD cluster into 15 distinct subfamilies. Interestingly, *Mycobacterium* ABC transporters may have undergone diverse evolutionary pathways, as homologues of the IrtB NBD are found in many subfamilies (Groups 15, 12, 4, and 5) (Figure 7). The specialized evolution of this transporter in pathogenic and non-pathogenic mycobacteria is highlighted by Group 15, which is unique to the *Mycobacterium* genus and contains the IrtB seeding query. Group 12 includes environmental species, including *Mycobacterium vanellbani* and *Mycobacterium gilvum*, suggesting that these organisms may have ecological adaptations unique to these species. IrtB clusters in Group 15 with members of the *Mycobacterium avium* complex, *Mycobacterium smegmatis*, and *M. tuberculosis* complex, supporting the notion that these clinically important mycobacterial species share an evolutionary ancestor [43].

In contrast, IrtA clusters in Group 4, notably close to a eukaryotic homologue, *Caenorhabditis briggsae*, and an iron transporter from *Methanosarcina acetivorans*, a versatile methane-producing archaea that thrives in diverse environments such as deep-sea hydrothermal vents and oxygen-depleted sediments beneath kelp beds (Figure 7). Given its unusual phylogenetic position, IrtA may have an old evolutionary origin that can be traced back to an archaeal predecessor. IrtA may have retained the ancestral features that support its function in the harsh and diverse conditions found within host macrophages, as shown by the strong link between *M. tuberculosis* IrtA and *Methanosarcina acetivorans* protein. These findings not only support the notion of a common evolutionary trajectory for members of the *M. tuberculosis* and *Mycobacterium avium* complexes but also point to the possibility of horizontal gene transfer events that shaped the functional evolution of these ABC transporters.

## 4. Discussion

For nearly all intracellular infections, including *M. tuberculosis,* iron acquisition is a crucial virulence factor [13,44,45]. To overcome the host’s iron sequestration mechanisms and establish a competitive dynamic at the host-pathogen interface, many pathogens rely on active iron transport systems. The capacity of the pathogen to successfully negotiate this vital barrier of protection determines its survival [46,47,48]. A deeper understanding of the physicochemical properties of these key biochemical components in pathogenesis is crucial for advancing therapeutic interventions. The present study makes significant inroads into the mechanistic features of the functioning of two ABC transporter systems of *M. tuberculosis*, shown by our previous study and subsequent other studies, to bring about siderophore-dependent iron uptake [12,14].

ABC transporters IrtA and IrtB-Rv2895c carry out siderophore-mediated iron uptake by utilizing their biophysical and biochemical properties, such as substrate-binding specificity and affinity, their unique structural make-up, and stability under varied pH and temperature ranges, and ATP hydrolysis-driven conformational changes, to actively transport unbound or iron-bound siderophores across the membrane. Their biomechanical properties, including the ability to undergo specific conformational shifts, enable efficient substrate recognition, binding, and precise directional transport, which include IrtA mediates export of unbound siderophores and IrtB-Rv2895c mediated import of iron bound siderophores. These properties are essential for maintaining iron homeostasis, particularly in the in vivo environments with limited iron availability.

Our findings demonstrate that ATP binding is essential for stimulating the homodimerization of the ATPase domains in IrtA and IrtB (Figure 3b,c and Figure 4c,d). However, only the ATPase I domain belonging to IrtA is capable of catalyzing ATP hydrolysis, suggesting that dimerization and activation of IrtA occur via the C-terminal ATPase end. This conclusion is further supported by the observation that the N-terminal SBD remains monomeric, regardless of whether it is in the carboxymycobactin-bound or unbound form (Figure 3a). The activity of ATPase I as an independent protein suggests that ATP hydrolysis initiates the signal for carboxymycobactin translocation to the extracellular space. The structural framework required for transmembrane domain (TMD) opening and subsequent translocation of carboxymycobactin may be provided by the conformational changes caused by carboxymycobactin binding in the SBD, which result in a more structured α-helix and a slight ordering of the initially less structured ATPase I upon ATP binding (Figure 4c). However, more experimental insight is needed to understand the primary conformational changes in the TMDs that result in their open, active conformation throughout the translocation process (Figure 4c). Further research is needed to elucidate the primary conformational changes in the TMDs that result in their open, active conformation during the translocation process.

The finding that ATPase II (linked to IrtB) may form stable dimers even in the absence of Na-orthovanadate raises the possibility that this dimeric state is preserved even after ATP hydrolysis, suggesting a possible locked or inactive conformation. However, further studies are necessary to elucidate the molecular function of ATPase II due to its unique stability, which suggests that it may activate IrtB through early ATP binding but may persist in a non-hydrolyzing form once dimerized. The two ATPases, namely, ATPase I of IrtA and ATPase II of IrtB, have different regulation mechanisms evidenced by the possibility that ATPase I needs signals other than ATP binding to activate. These results collectively point to a distinct activation strategy for IrtA and IrtB, where ATPase II may directly activate the transporter while ATPase I may rely on extracellular cues to accomplish full activity.

Additionally, the SBD domain of IrtA transports siderophores from inside the mycobacterial cell to the outside milieu. Its active monomeric nature suggests that it can independently bind siderophores with high specificity without the need for oligomerization (Figure 3a). Its monomeric nature also contributes to both the efficiency and adaptability of siderophore transport, supporting mycobacterial survival in iron-limited host environments. Monomeric characteristics of the active protein enhance flexibility and responsiveness to fluctuating iron supply within the macrophages. Furthermore, the active monomer structure might lower energy costs associated with multimeric assemblies, thereby enhancing transport efficiency. Overall, the monomeric structure of the SBD facilitates a streamlined and adaptable iron acquisition mechanism. Conversely, though ATPase II dimerizes in the presence of ATP, it does not hydrolyze ATP as an independent protein. The minimal secondary structural alterations seen in both ATP-bound and unbound forms indicate that it does not have the required stimulus to transition to an active conformation (Figure 3d and Figure 4d). We propose that the activation signal for ATPase II is provided by Rv2895c, which changes significantly from a completely α-helical structure to a large β-sheet following Fe-carboxymycobactin binding through the TMD of IrtB. This suggests that IrtB is activated when ferricarboxymycobactin binds to its TMD.

As seen in Figure 3b, the Rv2895c protein is a monomer in its substrate-unbound state. However, a combination of dimeric and monomeric species remains after substrate binding, even at a five-fold molar excess of substrate concentration, indicating a dynamic binding process (Figure 3b). In the presence of substrate, the dimeric form likely represents the substrate-bound state, while it reverts to a monomeric form upon substrate removal. Therefore, as previously shown, the interaction of substrate-bound Rv2895c with the TMD of IrtB would cause the conformational changes required to activate the ATPase, resulting in the active translocation of ferricarboxymycobactin [12]. The dynamic equilibrium between monomeric and dimeric forms of Rv2895c in the presence of ferricarboxymycobactin also suggests that substrate binding induces dimerization. The high stability of the ferricarboxymycobactin-Rv2895c complex at acidic pH, compared to that of the SBD, indicates its efficient functioning in internalizing iron-bound siderophores within the acidic environment of macrophage phagosomes (Figure 6b).

It is interesting to note that the SBD has a narrow pH activity range and comparatively higher temperature stability (Figure 6a), indicating that its minimal local pH changes may be required for its in vivo activity. Additionally, the stability of ATPase I at temperatures up to 60 °C and at pH levels as low as 4.0 may be attributed to the evolutionary adaptation of the IrtA protein from *Methanosarcina acetivorans*, an archaea capable of withstanding high temperatures and oxidative stress, as supported by phylogenetic analyses (Figure 5a,c and Figure 7) [49]. This archaea thrives in diverse environments, such as oxygen-depleted sediments under kelp beds and deep-sea hydrothermal vents [49]. The evolutionary linkage of IrtA with *Methanosarcina acetivorans* protein provides hints about the efficiency of IrtA. The transporter protein IrtA may have adapted for optimal functionality across the varied pH and temperature conditions encountered within host macrophages [50].

Changes in temperature have an impact on protein folding, kinetic energy of substrate binding, and stability. Temperature and pH variations have a substantial impact on the stability and functionality of the majority of transporter proteins, including the substrate binding of the IrtA and IrtB-Rv2895c transporters [51,52]. Iron-bound siderophores, which are vital for several physiological functions, including *M. tuberculosis* survival in a variety of host microenvironments, are mediated by the IrtA and IrtB transporters [53]. The ionization states of amino acid residues at the substrate binding site can be impacted by pH variations, which can alter the transporters’ affinity for substrates. Acidic or basic pH may cause non-specific conformational changes that decrease substrate binding efficiency or modify transport velocity.

The IrtA and IrtB-Rv2895c transporters are essential for bacterial survival, specifically in the context of *M. tuberculosis*, where iron acquisition is crucial for bacterial survival [11,12,14]. The pH and temperature settings these bacteria encounter within the host’s macrophages, where the environment is acidic and the temperature fluctuates at microlevels, are directly relevant to the function of the two transporters [54]. Analyzing the temperature and pH dependency aids in simulating the environmental stresses that these transporters encounter during infection and may identify therapeutically targetable vulnerabilities. These will offer a foundational basis for designing drugs/inhibitors and optimizing treatment processes.

It is plausible that the pH stability traits of IrtA and IrtB could be relevant to ABC transporters across infectious mycobacterial species. Many mycobacterial pathogens, besides *M. tuberculosis*, confront environmental problems in the acidic compartments of host macrophages, which is well-aligned with the increased stability of ABC transporter domains of IrtB at acidic pH (Figure 6a,b). Given this adaptability trait, it is possible that ABC transporters across different mycobacterial species involved in siderophore-mediated iron absorption likewise show comparable stability at low pH conditions.

Phylogenetic analysis of the two transporters, IrtA and IrtB, using the conserved NBD allows us to reduce background noise from the variable domains of the coding sequences, which might have different evolutionary histories. This approach leads to more consistent phylogenetic constructions. According to our results, IrtA is an intriguing example of horizontal gene transfer from an archaeal progenitor to prokaryotes and eukaryotes since it possesses the characteristics of an EK-type transporter and functions similarly to transporters of eukaryotic and archaeal origin (Figure 7). This finding is supported by the clustering of IrtA with a *Methanosarcina acetivorans* iron transporter, which is consistent with the earlier work that found IrtA to be a siderophore iron exporter (Figure 7) [12,55]. Additionally, the evolutionary association between IrtA and archaea suggests it’s resilient to changing microenvironments, a trait that may be shared by other pathogenic mycobacteria, which survive in a variety of physiological settings. These characteristics likely reflect widespread evolutionary adaptations among mycobacterial species, which enhance their pathogenicity and survival in a range of host microenvironments. Compared to earlier reports that mainly described IrtA and IrtB as multidrug transporters involved in cytochrome assembly, these findings mark a considerable development. Our mechanistic dissection of siderophore iron transporters and the phylogenetic studies reported here suggest a need for reclassification of *M. tuberculosis* ABC transporters.

## 5. Conclusions

In conclusion, our study provides a physiochemical and evolutionary analysis of the iron-siderophore export-import system in *M. tuberculosis*, emphasizing the conformational changes, pH, and temperature dependence of substrate binding and transporter activities. These findings will enable us to unravel important aspects of these transporters as drug targets. The two ABC transporters, IrtA and IrtB, are characterized by their distinct ATPase activities and substrate interactions, which highlight the crucial functions these proteins play in the iron acquisition strategy of the pathogen. The robustness of these transporters in a variety of environmental circumstances is further supported by phylogenetic studies that indicate an evolutionary adaption of these transporters, especially IrtA, from archaea like *Methanosarcina acetivorans*. However, our research highlights the potential of these transporters as therapeutic targets in addition to improving our knowledge of how they function in the varied microenvironment of the host macrophages. By elucidating the structural and functional dynamics of these transporters, we provide valuable insights that could guide the design of effective strategies to disrupt iron acquisition in *M. tuberculosis*, potentially leading to the development of novel anti-tuberculosis therapies.

## Data Availability

All data and materials used in this study are available with the corresponding author and will be provided upon reasonable request.
